# Predicting creative behavior using resting-state electroencephalography

**DOI:** 10.1038/s42003-024-06461-6

**Published:** 2024-06-29

**Authors:** Fatima Chhade, Judie Tabbal, Véronique Paban, Manon Auffret, Mahmoud Hassan, Marc Vérin

**Affiliations:** 1 CIC-IT INSERM 1414, Université de Rennes, Rennes, France; 2 Institute of Clinical Neurosciences of Rennes (INCR), Rennes, France; 3 MINDIG, Rennes, France; 4 CRPN, CNRS-UMR 7077, Aix Marseille Université, Marseille, France; 5 France Développement Électronique, Monswiller, France; 6 School of Science and Engineering, Reykjavik University, Reykjavik, Iceland; 7 B-CLINE, Laboratoire Interdisciplinaire pour l’Innovation et la Recherche en Santé d’Orléans (LI²RSO), Université d’Orléans, Orléans, France

**Keywords:** Cognitive neuroscience, Electroencephalography - EEG

## Abstract

Neuroscience research has shown that specific brain patterns can relate to creativity during multiple tasks but also at rest. Nevertheless, the electrophysiological correlates of a highly creative brain remain largely unexplored. This study aims to uncover resting-state networks related to creative behavior using high-density electroencephalography (HD-EEG) and to test whether the strength of functional connectivity within these networks could predict individual creativity in novel subjects. We acquired resting state HD-EEG data from 90 healthy participants who completed a creative behavior inventory. We then employed connectome-based predictive modeling; a machine-learning technique that predicts behavioral measures from brain connectivity features. Using a support vector regression, our results reveal functional connectivity patterns related to high and low creativity, in the gamma frequency band (30-45 Hz). In leave-one-out cross-validation, the combined model of high and low networks predicts individual creativity with very good accuracy (r = 0.36, p = 0.00045). Furthermore, the model’s predictive power is established through external validation on an independent dataset (N = 41), showing a statistically significant correlation between observed and predicted creativity scores (r = 0.35, p = 0.02). These findings reveal large-scale networks that could predict creative behavior at rest, providing a crucial foundation for developing HD-EEG-network-based markers of creativity.

## Abstract (web summary)

Combining HD-EEG with connectome modeling predicts individual creative behavior from resting-state functional connectivity, highlighting the importance of optimal activation interplay across networks in creative cognition.

## Introduction

Creativity plays a fundamental role in shaping our cultural and technological landscape. It has consistently been the driving force behind artistic, inventive, and scientific endeavors, fueling innovation and pushing the boundaries of human progress. As the world becomes increasingly complex, the demand for creativity continues to grow, not only in the ability of innovation and problem-solving but also as a dynamic response to the ever-evolving nature of human existence. This multifaceted significance of creativity has led to extensive research in various disciplines, attracting particularly the interest of the neuroscience community^1^. However, the cognitive mechanisms underlying creative abilities are not yet fully understood.

Over the past years, emerging evidence showed that complex brain functions like creativity are generated by large-scale networks of highly specialized and spatially segregated brain regions^2^ i.e., functional connectivity. Motivated by the enormous progress that has been made in developing neuroimaging tools, we have witnessed substantial advancements in functional connectivity analysis on creativity. Functional connectivity quantifies the temporal dependencies between regions and networks, enabling us to investigate the network organization of the human brain^3^.

Traditionally, functional connectivity studies primarily focused on group-wise differences in clinical and healthy populations and decoding functional connectivity patterns associated with specific cognitive states. In this context, inter-individual variability was often viewed as a potential source of noise. However, recent research has demonstrated that functional connectivity patterns can be unique to individuals and exhibit a relatively stable nature across different cognitive states, be it during task performance or rest^4–7^. These patterns have been successful in predicting individual traits, cognitive behavior, and clinical features, resulting in the rise of a new dimension of personalized neuroscience focused on behavioral prediction^8–16^.

Connectome-based predictive modeling (CPM)^10^ leverages the most relevant features of functional connectivity to predict behavioral outcomes. By mapping the brain’s intricate connections and integrating them with data on individual behaviors, it offers a window into the neural basis of highly complex phenomena. This emphasized the importance of considering individuality and variability in the study of brain networks related to the complex processes of creativity. Consequently, task-based and resting-state functional connectivity (RSFC) measured by functional magnetic resonance imaging (fMRI), within and between brain networks, were mapped to predict individual differences in creative abilities^9,10,17–21^. These researchers considered the advantage of a whole-brain functional connectivity approach over localization-based or between-group approaches to provide a functional view of how brain networks relate to individual creative ability. Moreover, predictive modeling approaches were built with integrated cross-validation, which is a powerful method to estimate a brain-behavior relationship^22^. Crucially, it facilitates testing the strength of the relationship in novel observation via both internal and external validation, permitting to establish the generalizability of the findings to independent datasets.

Based on that, whole-brain networks associated with creative abilities have been uncovered to support a growing body of creativity research that highlights the importance of functional interactions within and between multiple brain networks, including the executive control network, the salience network (SN), and the default mode network (DMN)^17,23^. However, these studies have generally classified creativity based on creative task performance (e.g., divergent thinking) rather than individual differences in real-life creative behavior. Divergent thinking measures creative potential, which refers to an individual’s inherent ability for creativity. Creative behavior on the other hand, also referred to as real-life creativity, is the tangible outcome of applying one’s creative potential in the real world, which reflects the individual differences in cognitive ability but also environmental factors and personality ^24^.

In the current paper, we aim to show how computational network neuroscience can be used to couple cognitive and neural analysis on a behavioral level. A recent study has applied connectome predictive modeling to explore real-life creativity by studying the neural basis of semantic memory organization related to creative behavior^25^. Using functional connectivity measured from acquired fMRI data while participants underwent a semantic relatedness judgment task, the team identified patterns of task-based functional connectivity that predicted creativity-related semantic memory network properties.

While all these studies have focused on modeling specific task-related functional connectivity measured by fMRI, the present study explores the EEG resting-state (RS-EEG) network determinants of real-life creative behavior.

The recognized success of functional connectivity research has inspired a growing interest in analyzing brain functional networks using EEG, as a practical, easy-to-use, and relatively low-cost neuroimaging technique. In contrast to fMRI, EEG reflects a direct measure of neural activity and allows the computation of brain oscillations within specific frequency bands. These oscillations may reflect important properties of network interactions at local and large-scales^26^. When combined with source reconstruction approaches^27^, EEG can be implemented to study functional interactions among cortical regions. Several studies have used EEG to investigate functional patterns associated with creativity, during creative thinking^28–31^ or at rest^32^. However, very few studies have coupled EEG with machine learning methods like CPM to explore how individual creativity influences functional connectivity.

Using HD-EEG in our research would offer an important extension to the literature on functional connectivity basis of creativity in extending CPM methods to EEG and the research of real-life creativity. We hope that integrating the HD-EEG more into these types of analyses would not only provide insights into the generalizability, sensitivity, and reproducibility of brain findings but also encourage exploiting the exceptionally high temporal resolution of this tool (millisecond compared to second in fMRI) and integrating more dynamic features in connectome analyses in future studies.

Therefore, in the present research, we applied the CPM approach^10^ to examine how whole-brain RS-EEG functional connectivity patterns could predict individual creative behavior and we hypothesized the presence of a stable resting-state neurophysiological marker of real-life creative behavior. Furthermore, we conducted an external validation analysis to establish the generalizability of the resulting neural model to an independent EEG dataset.

## Results

### Predictive networks of creativity

HD-EEG data was acquired from healthy adult participants during an eyes-closed resting state. All participants completed a creativity questionnaire (Inventory of Creative Activities and Achievements Questionnaire -ICAA-); which is a broad-based assessment of individual differences in real-life creativity. The inventory provided a *creative activity* score (C act), a *creative achievement* score (C ach), and a *general creative behavior* score (C total). In this study, we aimed to discover the existence of EEG networks that can predict the general ICAA creativity scores. Thus, whole-brain functional networks were constructed for each participant by computing the source-space functional connectivity among 68 brain regions of interest, in five frequency bands (delta, theta, alpha, beta, and gamma). A correlation between all functional connections and creativity scores, followed by a statistical threshold (*p* < 0.01), was used to identify which functional edges are significantly related to creativity in each frequency band. To determine which of these networks have a predictive potential, we used the connectome-based predictive modeling approach (CPM)^10^, where leave-one-out cross-validation (LOOCV) was performed to build and test network-based predictive models (i.e., internal validation). The analysis detected no relevant predictive networks of creativity in the delta, theta, alpha, or beta bands (Supplementary Table 1).

**Fig. 1:**
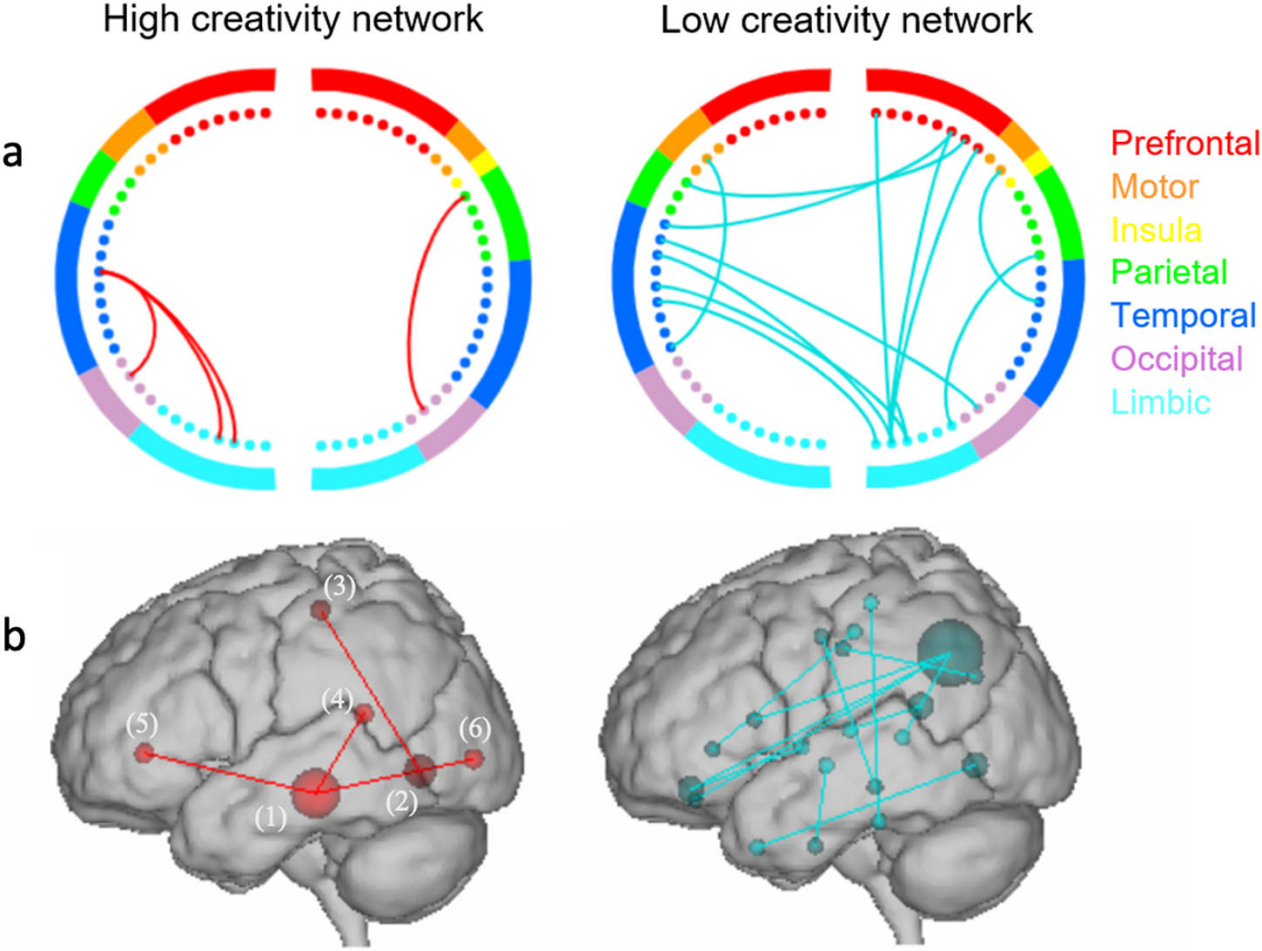
Depictions of high and low creativity networks. **a** Circle plots and (**b**) glass brains of high and low creativity networks. Colors within the circle plots correspond to brain lobes. **b** (1): the right middle temporal gyrus, (2): the lingual gyrus, (3): the left paracentral lobe, (4): the isthmus of the cingulate gyrus, (5): the rostral anterior cingulate cortex, (6): the lateral occipital cortex.

In the gamma band, it revealed a “high-creativity network” consisting of 8 edges positively correlated with ICAA scores, and a “low-creativity network” consisting of 26 edges negatively correlated with ICAA scores (total possible edges of 2278**)**. These predictive networks were consistent among 90% of all LOOCV folds. The high-creativity network exhibited dense functional connections among 6 regions: the right middle temporal gyrus (associated with semantic and working memory), 3 regions within the DMN (lingual gyrus, rostral anterior cingulate cortex, and the isthmus of cingulate gyrus), 2 within the visual association network (the lateral occipital cortex, the lingual gyrus is involved in the visual association network as well), and 1 within the somatomotor network (the left paracentral lobe). The low-creativity network showed diffuse connections among 20 regions across the whole brain. 6 were within the DMN, 4 were within the sensorimotor network (SMN), 2 within the frontoparietal network, 2 within the ventral attention network, 1 within the visual network (VN), and 5 temporal structures (Fig. 1).

### Internal validation: prediction of creativity from RS-EEG data

As internal validation, we followed a LOOCV analysis. This step aims to test the predictive performance of the brain connectivity-based models that reflect the strength of functional connectivity within the high and the low creativity networks, respectively. In the leave-one-out loop, 90 rounds (i.e., number of participants) of cross-validation were performed, during which, one different participant was left out from building the model each time, then used to test the model performance. This caused slight differences in networks in each round, as well as the model and its performance. Thus, the final evaluation of the model was the average performance across the 90 folds.

**Fig. 2:**
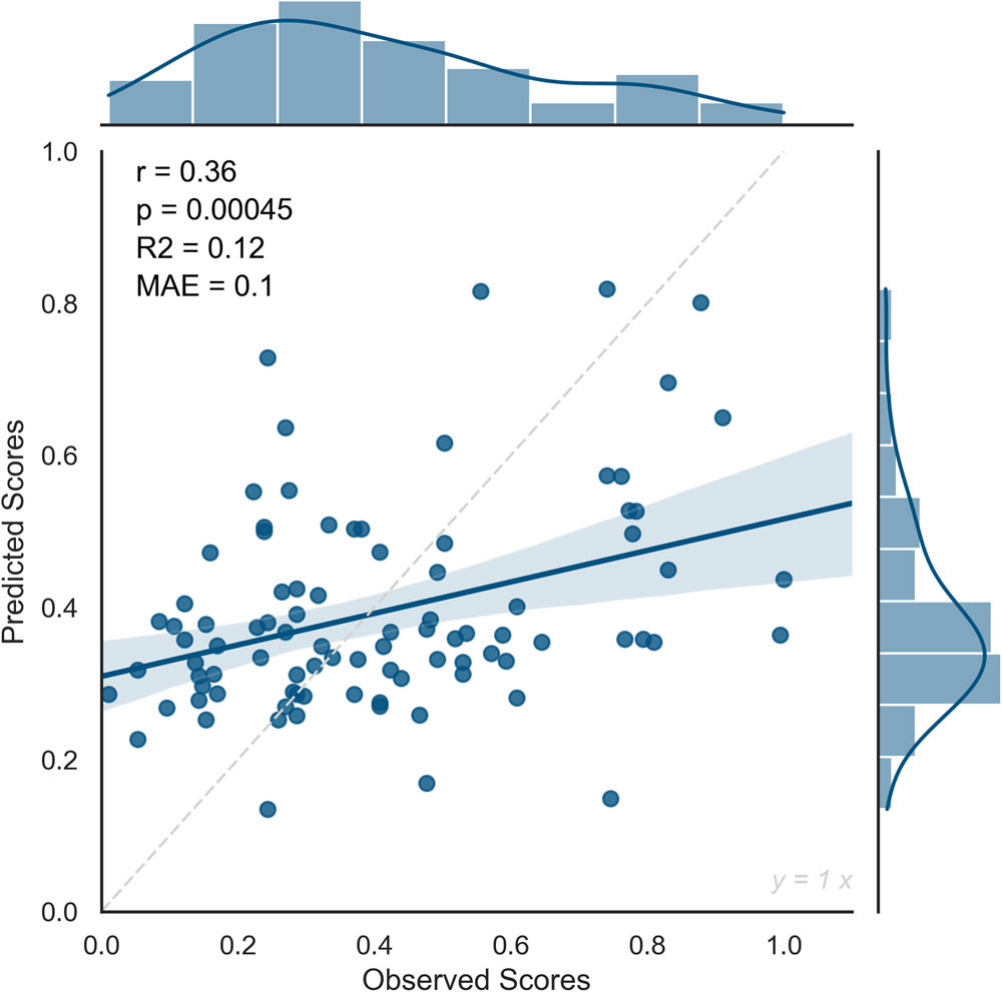
The internal validation of the CPM model. The relationship between observed and predicted creativity scores in the leave-one-out cross-validation of the CPM model, showing Pearson’s correlation coefficient (*r*), the *p* value (*p*), the *R*-squared (*R*^2^), and the Mean Absolute Error (MAE). *N* = 90 healthy participants.

We evaluated the predictive power of the model by assessing the statistical significance of the relationship between the observed and the model-predicted creativity scores. Results showed that high or low-creativity models alone could not robustly predict creativity. But interestingly, the combination of both networks could reliably predict real-life creativity (*r* = 0.36, *p* = 0.00045, MAE = 0.1, *R*-squared = 0.12) (Fig. 2). Additionally, a non-parametric permutation test of ICAA scores (5000 times) was added to ensure that the obtained correlation is significantly better than expected by chance. The permutation test results confirmed the significance of the combined network (*p* = 0.02). This internal validation demonstrates that individual differences in real-life creativity could be predicted from the strength of RS functional connectivity within the combination of both high and low-creativity networks.

### External validation: prediction of creativity using novel RS-EEG data

Furthermore, to strengthen the generalizability of our predictive model, we conducted an external validation analysis using a second independent RS-EEG dataset.

**Fig. 3:**
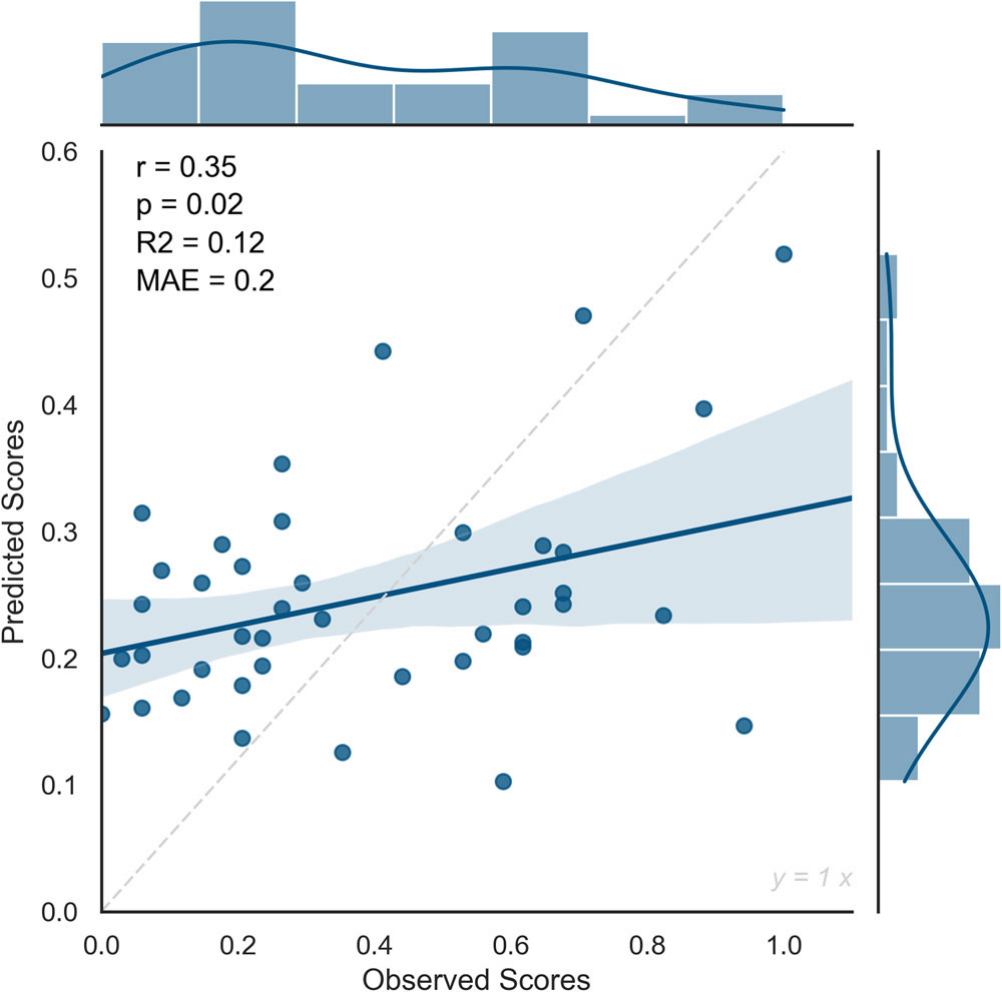
The external validation of the CPM model. The relationship between observed and predicted creativity scores in the external validation of the CPM model, showing Pearson’s correlation coefficient (*r*), the *p* value (*p*), the *R*-squared (*R*^2^), and the Mean Absolute Error (MAE). *N* = 41 healthy participants, independent sample.

High and low-creativity network strength values were thus computed for each subject in the new dataset. Then we used the trained model derived from the internal validation procedure to predict their creativity scores. Results revealed a significant predictive performance of the model (*r* = 0.35, *p* = 0.02, MAE = 0.2, *R*-squared = 0.12) (Fig. 3).

This external validation generalized the combined creativity model to a novel and distinct sample, indicating that participants with higher ICAA scores showed stronger functional connectivity within the high-creativity network and lower functional connectivity within the low-creativity network.

### Control analysis

Our study’s primary objective was to develop a predictive model for general creative behavior using the total ICAA score as the main outcome measure. However, we conducted a thorough control analysis using C act and C ach separately, in order to discern whether the model is predominantly influenced by one or the other. Analyses revealed that the model’s performance was driven by the achievement score more than the activity score (Table 1).

**Table 1:** Control analysis of the predictive model using creativity scores separately

	Int. Val	Ext. Val
	Combined net	Combined net
C act	*p* = 0.003, *r* = 0.30	*p* = 0.1
C ach	*p* = 0.0005, *r* = 0.35	*p* = 0.01, *r* = 0.39
C total	*p* = 0.00045, *r* = 0.36	*p* = 0.02, *r* = 0.35

Correlation coefficient (*r*) and *p* value (*p*) of the relationship between observed and predicted creativity scores in the internal validation (Int. Val) (*N* = 90 healthy participants), and the external validation (Ext. Val) (*N* = 41 healthy participants). Validation processes were done for the combined network (net) first using the general creativity score (C total), then using the creative activity (C act) or the creative achievement (C ach) scores separately.

### Cross-validation - LOOCV vs. k-fold

Cross-validation stands as a primary advantage of the CPM methodology, where it evaluates the predictive performance of the connectome models. Generally, this step can be achieved using two main approaches of cross-validation, (1) a k-fold cross-validation in which the data are split into k different subsets or folds, and (2) leave-one-out cross-validation (LOOCV), which was selected and initially performed in our analysis. LOOCV is the simplest form and the most popular choice, employed consistently in creativity research. However, many researchers discussed the merit of the k-fold method as well, considering that it gives less variable estimates of the prediction error than those from LOOCV^33^. In pursuit of methodological validation, we implemented the k-fold cross-validation technique, using 5 and 10 folds, to thoroughly assess the consistency and reliability of our models. Results did not yield the same statistical significance as the findings derived from LOOCV processing (Table 2).

**Table 2:** Cross-validation: LOOCV vs. k-fold

*P* Values of The Internal Validation			
Network	LOOCV	5 Folds	10 Folds
Positive	0.06	0.12	0.2
Negative	0.01	0.09	0.2
Combined	0.00045	0.09	0.3

The differences in *p* values resulting from applying Leave-one-out cross-validation (LOOCV) or K-folds (5 and 10 Folds) methods in the internal validation process, in the gamma frequency band. *N* = 90 healthy participants.

### Dynamic functional connectivity

In the pursuit of leveraging the high temporal resolution of HD-EEG and testing its further capabilities in connectivity analyses, we employed a dynamic approach within the same frequency of the static predictive modeling described above (the gamma frequency band). The approach consisted of quantifying the variance of the dynamic connectivity matrices for each subject over time (see methods). Then, we applied the CPM method on the resultant variance matrix to investigate the presence of dynamic-dependent predictive features of creativity. Results revealed no significant predictive model of either the creative activity, the creative achievement, or the general creative behavior in the gamma frequency band.

**Fig. 4:**
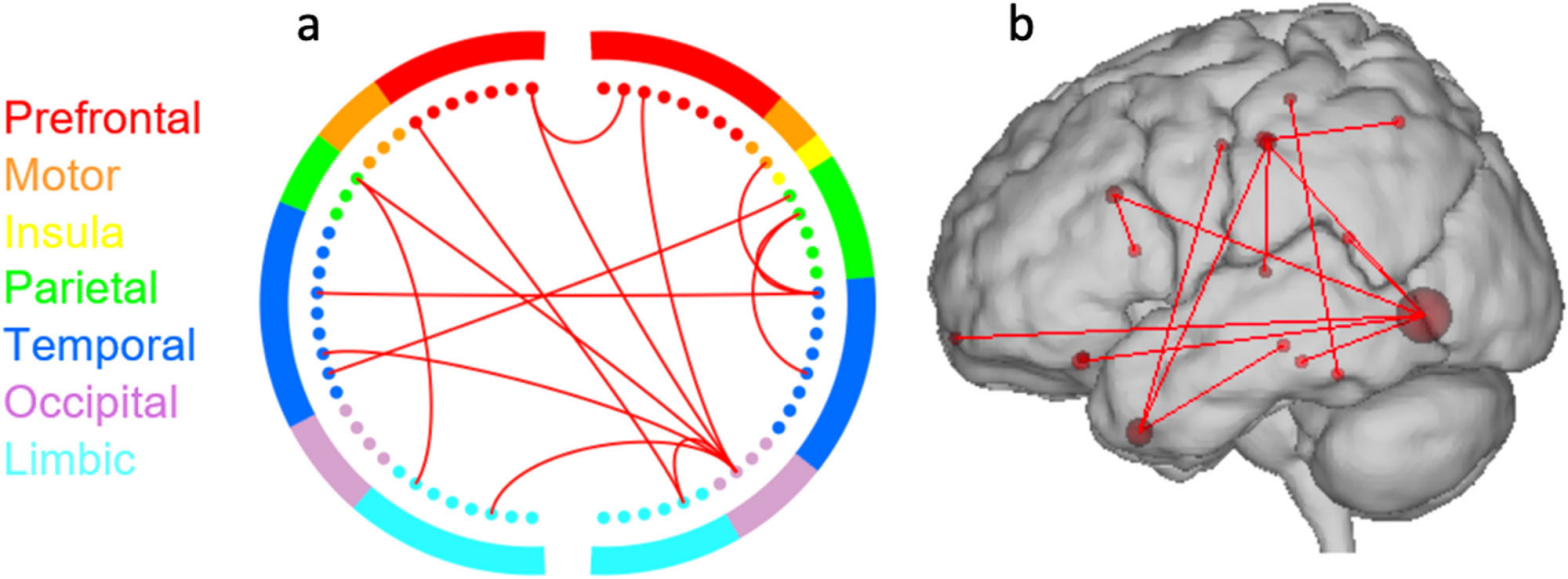
Depictions of high creativity network of dynamic connectivity analysis. **a** Circle plot and (**b**) glass brain of the high creativity network. Colors within the circle plots correspond to brain lobes. The network was consistent across 99% of iterations within the cross-validation loop.

Nevertheless, our dynamic analysis revealed a predictive model of creative achievement based on a high-creativity network in the delta frequency band. The high-creativity network exhibited dense functional connections among 17 regions: the right middle temporal gyrus (associated with semantic and working memory) and 3 other temporal structures (the right fusiform gyrus, the left temporal pole, and the Left transverse temporal gyrus), 4 regions within the DMN (lingual gyrus, the left caudal anterior cingulate cortex, the right Para hippocampal gyrus, and the right isthmus of cingulate gyrus), 1 within the SN (the right pars opercularis), 3 prefrontal structures (the left frontal pole, the left lateral orbitofrontal cortex, and the right lateral orbitofrontal), 3 within the somatomotor network (the right postcentral gyrus, the left paracentral lobe, the left postcentral gyrus, and the left precentral gyrus), 1 within the visual association network (the lingual gyrus), and 1 within the dorsal attention network (the right superior parietal lobule) (Fig. 4).

**Fig. 5:**
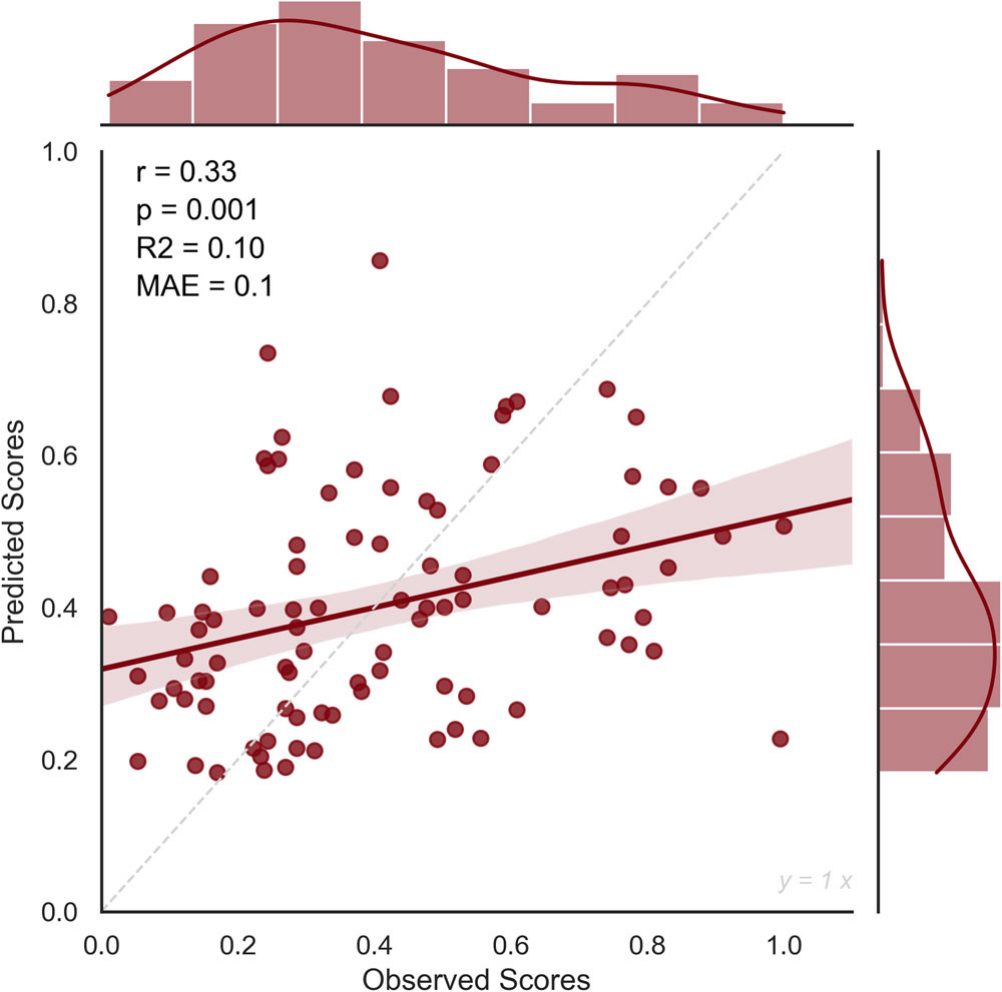
The internal validation of the dynamic connectivity model. The relationship between observed and predicted creativity scores in the leave-one-out cross-validation of the CPM model, showing Pearson’s correlation coefficient (*r*), the *p* value (*p*), the *R*-squared (*R*^2^), and the Mean Absolute Error (MAE). *N* = 90 healthy participants.

**Fig. 6:**
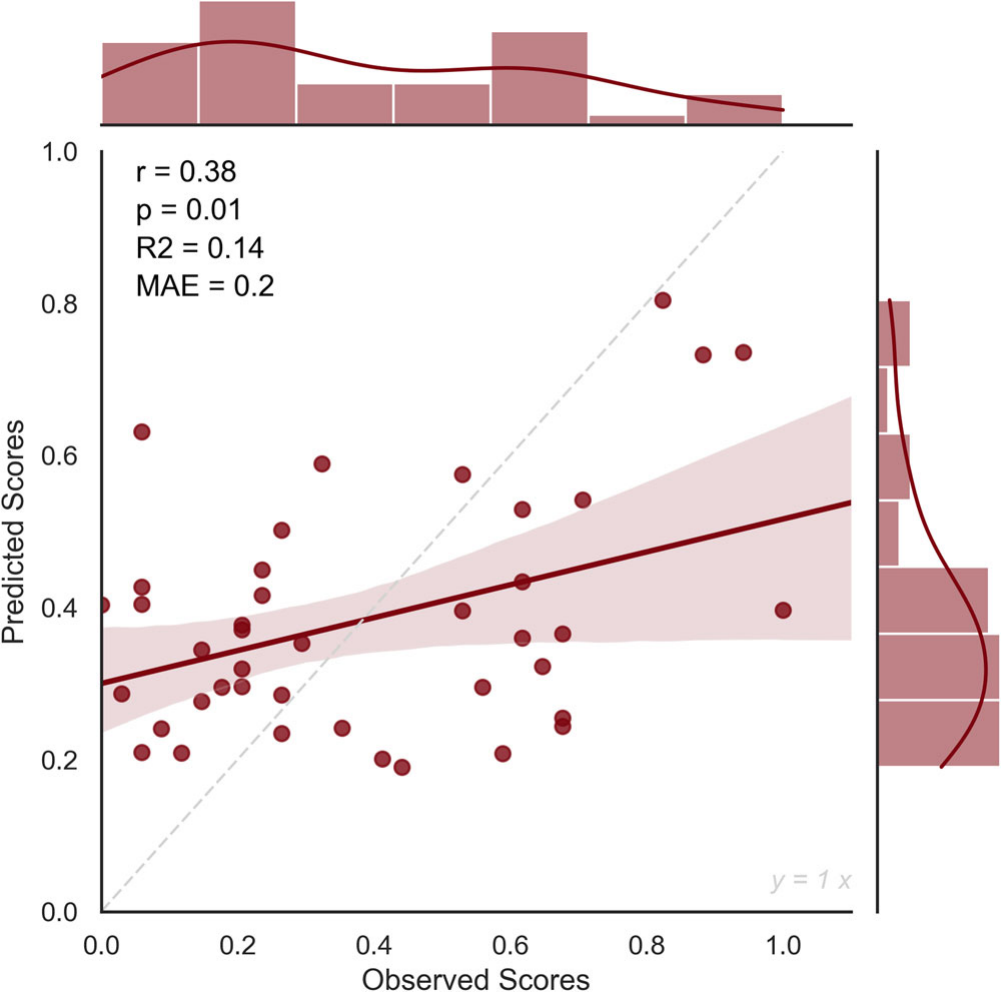
The external validation of the dynamic connectivity model. The relationship between observed and predicted creativity scores in the external validation of the CPM model, showing Pearson’s correlation coefficient (*r*), the *p* value (*p*), the *R*-squared (*R*^2^), and the Mean Absolute Error (MAE). *N* = 41 healthy participants, independent sample.

In a LOOCV, the network predicted C ach with an *r* = 0.33 and a p-value of 0.001 (Fig. 5). Additionally, the permutation test resulted in a *p* value of 0.04. Likewise, in an external validation process using the second independent dataset, the model predicts C ach scores in novel subjects, with an *r* = 0.38 and a *p* value of = 0.01 (Fig. 6). These findings highlight the complementarity of dynamic properties in the functional architecture of the creative brain.

### Confounder analysis

To explore potential confounding variation, we correlated each available co-variable (age, gender, education) with the creativity score. Results showed no significant relation with neither age (*p* = 0.06), gender (*p* = 0.7), or education (*p* = 0.36). Consequently, no cofounder effect was detected and thus considered as a variable in the predictive model.

## Discussion

Using HD-EEG and a machine learning technique, we established a connectome-based model capable of predicting individual real-life creativity. The model is based on resting-state networks associated with high and low creativity, respectively. Critically, the model demonstrated its generalizability by successfully predicting creativity scores across an independent EEG dataset. These results indicate that creativity may be captured not only by task-induced variation of functional connectivity^17,25^ but also by stable trait-level variation of intrinsic functional connectivity at rest. Several researchers have examined RSFC and have revealed consistent RSFC large-scale networks in the human brain e.g., DMN, VN, SMN, cognitive control network^3,34,35^, and SN^36^. Recent findings suggested that creativity can be associated with the strength of FC among specific patterns of interactions between brain regions of those resting-state networks^19^. Our results align with these findings and increase evidence that real-life creative behavior can be predicted from an individual’s resting EEG connectivity profile.

The high-creativity network exhibits functional connections among components of the DMN, which is involved in constructing dynamic mental simulations based on the past, the future, and imagination^37^. Consistent with our findings, DMN was largely associated with creativity. The DMN activity has been positively correlated with higher creativity at rest, as divergent thinking ability ^38^ and verbal creativity ^39^. Moreover, DMN regions are often involved in task-induced creativity such as visual creativity^40^, creative story generation^41^, and insightful problem-solving^42^. In addition to the default mode, the high creativity network included the middle temporal gyrus tightly associated with semantic memory and processing^43^ and core regions of the visual association network. Visual association is necessary to identify objects and interpret the same object across different specific contexts^44^. Evidence showed that visual association is closely associated with memory performance^45–48^. Recent work suggested an important role for visual association areas in associative memory^49^—the ability to bind together previously unrelated information —underlies the formation of episodic memories^50^. The engagement of DMN, semantic, and associative ability is in line with associative theories of creativity. Particularly, the episodic memory theory; the constructive episodic simulation hypothesis^51^. The theory postulates that memory and imagination, together, implicate flexible recombination of episodic memories such as places, people, objects, and details of events. This could be very interesting since, in part, creativity is supported by our abilities to recall memories and envision the future, especially at rest. This flexible nature of episodic processes seems to be particularly involved in creativity, which requires connecting both memory and imagination in new, original, and meaningful ways. This builds on other works that have indicated that episodic memory is closely related to creativity^52,53^, and the predictive model of functional connectivity relating real-life creative behavior to semantic memory ^25^. While this latter study focused specifically on the semantic basis of creative cognition and its relationship with task-based connectivity patterns, our study took a broader approach by considering the overall RSFC and incorporating an external validation process. However, despite the methodological differences, both results converge on the importance of associative abilities in predicting real-life creativity. Furthermore, the identification of the default DMN, somatomotor, and visual associative areas within the high creativity network in both studies implicated significantly these networks in supporting real-life creative behavior. This highlights the role of semantic memory and associative brain regions in creative behavior and underscores the significance of associative abilities within our predictive networks. This alignment between the two models suggests a robust association between associative processing and creative behavior.

The low-creativity network, on the other hand, exhibits diffuse functional connections among several resting state networks, especially DMN and SMNs, in addition to temporal structures. Although DMN regions are associated with high creative ability, default activity was also linked with prepotent response tendencies^54^ and strong semantic associations^55^. Additionally, sensorimotor regions were found to support procedural previously learned information^56^. This could suggest that low creativity may be characterized by increased interactions among regions that support memory, and automatic common associations and representations that are in turn, not effectively regulated by high-creativity brain regions.

In our results, neither high creativity nor low creativity networks alone could predict robustly individual creativity. But interestingly, together, they construct an effective predictive model. Specifically, higher activation of the high creativity network coupled with lower activation of the low creativity network correlates with higher levels of creativity in individuals. This suggests that individuals who predominantly engage the brain regions associated with semantic memory and associative abilities (high creativity network) while simultaneously minimizing activation of regions involved in other cognitive processes (low creativity network) tend to exhibit greater creative behavior.

Furthermore, the low-creativity network involves the prefrontal cortex, whereas the high-creativity network does not. Given the observed inverse relationship between the activation levels of the high and low creativity networks at rest, this could propose that while activating the high creativity network is crucial for facilitating creative behavior through semantic memory and associative processing, excessive activation of the low creativity network, which includes regions like the prefrontal cortex typically associated with cognitive control and regulation, may hinder the creative process at rest. Therefore, creative behavior may necessitate a delicate balance between engaging the high creativity network to foster creative performance and attenuating the activation of the low creativity network to prevent over-regulation or inhibition of creative processes. This hypothesis underscores the intricate interplay between different neural networks in creative cognition at rest and highlights the importance of optimal activation levels across these networks for maximizing creativity. However, further empirical research is needed to fully elucidate the mechanisms underlying this balance and its implications for creative performance.

Our statistical analysis showed that only the gamma frequency band highlighted a robust internal and external predictive network of high creative behavior, and no other significant outcomes were found in other EEG frequency bands (Refer to Supplementary Fig. 1 for power spectral density information). These observations may be explained by the fact that among all frequencies, the gamma band (30–100 Hz) in EEG is most closely associated with high-order cognitive function^57^. It was shown to reflect multiple cognitive processes such as attention, language, binding, and object representation^58^. Interestingly, rapid oscillatory activity in the gamma band has been identified as a fundamental mechanism for effectively encoding and retrieving episodic memories^59,60^. Furthermore, it was suggested that gamma band activity is involved in creative thinking^61^, and plays a critical role in creative insights, as was demonstrated by a study where EEG recordings revealed a burst of gamma activity beginning shortly prior to insightful solutions for verbal problems^42^. However, when examining neural mechanisms underlying creativity using EEG, the gamma band wasn’t the only frequency band that highlighted creative ability. Alpha band activity on the other hand was reported to correlate with divergent thinking ability^62–65^. While there is a general assumption of the involvement of alpha band activity in creative thinking processes, inconsistent results have been reported for alpha and beta bands. This inconsistency can be attributed to the heterogeneity of employed methods and participant samples as well as the relatively limited number of studies investigating EEG correlates of creativity.

From another point of view, there is an assumption that high frequencies like the gamma band are associated with local information processing (segregation) while low frequencies (theta, alpha) are associated with global information processing (integration). However, connectivity-based studies have linked high gamma frequency with long-range connections and network integration associated with cognition and behavior at resting state. This apparent contradiction may be related to the difference in the methodological approaches and between the localization versus connectivity-based analyses.

Moreover, numerous functional connectivity studies have examined the statistical associations (i.e., correlations) between creativity and brain connectivity. Some of these studies have misinterpreted these associations as predictions. Yet, significant correlation within a sample does not necessarily indicate predictive ability^66^. Thus, the advantage of this work lies in its use of cross-validation to rigorously assess the presence of a resting state FC-creativity relationship. By evaluating the predictive power of creativity networks, this approach provides a robust test of this relationship. Generally, there are two approaches of cross-validation, (1) a k-fold cross-validation in which the data are split into k different subsets or folds, and (2) leave one out cross-validation (LOOCV). LOOCV is consistently employed in the predictive modeling of creativity and thus was performed in our analysis. However, many researchers discussed the merit of the k-fold method, considering that it gives less variable estimates of the prediction error than those from LOOCV^33^. Therefore, as a methodological validation, we implemented the K-fold cross-validation technique to thoroughly assess the consistency and reliability of our models and test K-fold performance. Results showed no consistency between the two methods and significantly better performance of LOOCV in our data. Nevertheless, the robustness of our LOOCV-derived model was largely due to the complementary external validation that allowed our experimental design to be particularly powerful and our model to be generalized to an independent dataset.

Another consideration in this study is that we used a self-reported questionnaire—the ICAA questionnaire—to assess participants’ creativity. The questionnaire provides scales for the frequency of engagement in everyday creative activity and the level of creative achievement across the most common eight creative domains. It offers a broad assessment and a quick gathering of a large amount of information on participants’ real-life creative behavior across multiple domains and levels. We ensured that participants completed the questionnaire in private with the assurance of confidentiality and anonymity to promote more truthful and accurate responses. Nonetheless, like all self-reported data collection, they are subject to limitations of honesty and reliability, given that people tend to be consciously or unconsciously biased when they report on their own experiences. However, the reliability and validity of the ICAA scores have been supported by a rigorous formal test analysis conducted on a large sample size of 1556 individuals^67^.

In the end, it is crucial to acknowledge that studying creativity poses a major challenge due to its multifaceted and complex nature, which manifests in various forms and engages multiple brain regions. Unlike other aspects of cognition that have been attributed to localized brain activity, creativity is a network phenomenon that involves complex neural interplay between multiple brain regions across the whole brain. Considerable further research is needed to uncover the diverse manifestations of creativity and the distinct roles of different networks in the creative brain. Network-based approaches, such as connectome-based modeling, provide promising tools to address these questions. However, it is important that future researchers continue to further explore network dynamics underlying creative processes. As our dynamic functional connectome results illustrated, employing the CPM method on HD-EEG data could offer deeper insights into the dynamic network properties associated with creativity. The association between EEG functional connectivity and creativity seems to vary depending on the frequency and approach employed. Therefore, it merits comprehensive exploration across diverse paradigms and contextual frameworks.

## Methods

### Participants

#### Two independent datasets have been used for the study

Dataset 1 was collected as part of the current study. A total of 98 healthy participants were recruited at the University Hospital Centre of Rennes from local and surrounding communities (60 female, mean age = 39.6 y ± SD = 12.7). Flyers for a call for voluntary participation were digitally and locally distributed, on Twitter, LinkedIn, and Facebook groups, and in universities, art and dance schools, cultural centers, libraries, etc… Participants from various creative domains such as art, dance, music, and sciences were selected to ensure a diverse population. All participants were French native speakers, aged between 18 and 68 years, and with normal or corrected vision. They reported no situation or history of cognitive disability, neurological disorders, or medication that can affect the central nervous system. All ethical regulations relevant to human research participants were followed. The study was approved by the ethical committee of the University Hospital Centre of Rennes (agreement n°20–171) and each participant provided written informed consent.

Dataset 2 was part of a different study at the University of Marseille in France. It consists of 52 healthy participants (28 female, mean age = 45.8 y ± SD = 17.3). The same inclusion criteria were applied. The study was approved by the “Comité de Protection des Personnes Sud Méditerranée” (agreement n°10–40) and each participant provided written informed consent prior to acquisition.

### Behavioral assessment

Creativity was assessed by the verified French version of the “Inventory of Creative Activities and Achievements” questionnaire (ICAA): a broad-based assessment of individual differences in real-life creativity^67^. The questionnaire provides independent scales for the frequency of engagement in everyday creative activity and the level of creative achievement across 8 creative domains (i.e., literature, music, arts and crafts, creative cooking, sport, visual arts, performing arts, and science and engineering). For each participant, two scores were calculated: the creative activity score (Cact) based on the frequency of engagement in everyday creative activity, and the creative achievement score (Cach) based on the level of publicly acknowledged creative achievement. In this study, we used the total creativity score by summing Cact and Cach to obtain one behavioral score for each participant (C total). The total score can range between 1 (for least creative) and 472 (for most creative).

It is pertinent to highlight that Dataset 2, sourced from Aix-Marseille University, exhibited some variations in the calculation of Cact and Cach scores compared to the standard methodology outlined in the reference inventory. Despite applying different scales, the differentiation between responses was maintained, thus preserving the graduating trend of the evaluation process. Our analysis revealed consistent patterns, wherein similar subjects consistently exhibited lower or higher scores throughout the evaluation. Nevertheless, in order to enhance the consistency and reliability of our analyses, we implemented normalization adjustments before conducting the analyses to address the differences within the shared dataset. This normalization process involved scaling the scores to a uniform range between 0 and 1. This was achieved through division by the maximum value observed within the score’s dataset.

### EEG data acquisition

EEG recordings were collected during a resting state. During the acquisition session, participants were seated comfortably in a dimly lit room and asked to rest and relax for 5 to 6 min while closing their eyes without falling asleep. In Dataset 1, the EEG was acquired with the high-density 256-channel HydroCel Geodesic Sensor Net (Electrical Geodesics Inc., Eugene, OR, United States) at a sampling rate of 1000 Hz. We referenced all electrodes to Cz, and impedances remained below 50 kΩ. In Dataset 2, EEG recordings were collected using a 64-channel Biosemi ActiveTwo system at a sampling rate of 2048 Hz.

It is noteworthy that the inventory of creativity was completed either in several days in advance of the EEG recording session, for participants who had been previously contacted, or after the session for those who had not completed it beforehand. To mitigate potential confounding factors, we deliberately avoided scheduling respondents to complete the inventory before the recording session on the same day. This precautionary measure was implemented to minimize the likelihood of any influence stemming from the administration of the questionnaire on the resting state recording.

### EEG data preprocessing

We applied a semi-automated preprocessing protocol for both datasets. First, for each participant, 5–6 min signal was segmented into non-overlapping 40-s epochs. The epochs of Dataset 2 were resampled at 1000 Hz to equalize the sampling frequency. Then, we used an automatic protocol on AUTOMAGIC®; an open-source MATLAB-based toolbox for EEG preprocessing^68^. The protocol consisted of three main steps: (i) bad channel identification, (ii) artifact correction including a pass filter between 1 and 45 Hz and EOG regression, and (iii) interpolation of detected bad channels using neighboring electrodes within a 4–5 cm radius. Furthermore, to ensure good signal quality, each epoch was visually inspected, and additional bad channel detection and interpolation were performed as required. After interpolating, we applied a 15% threshold, meaning the exclusion of every epoch that needed more than 15% of electrodes to be interpolated. Finally, we selected three artifact-free epochs of 40 s in length for each participant. Due to their poor signal quality, 8 participants were excluded from the first dataset and 11 from the second. Bad quality was determined based on quantified criteria; either an excessive amount of needed interpolation and/or high muscle artifact.

### Brain network construction

Functional brain networks were estimated using the “EEG source connectivity” method^2^, combined with a sliding window approach^69^. The method includes two main steps: (i) solving the EEG inverse problem to estimate the cortical sources to reconstruct their temporal dynamics and (ii) measuring the functional connectivity between the reconstructed scout time series. In order to solve the inverse problem, the weighted minimum norm estimate (wMNE) algorithm^70^ was used to estimate regional time series between predefined regions of interest (ROIs). To define ROIs, we used the Desikan Killiany atlas, which parcellates the cortical surface into 68 ROIs^71^. The functional connectivity between the 68 regional time series was then obtained using the phase-locking value metric (PLV). The combination of (wMNE/PLV) proved its efficiency in identifying the cortical brain networks from scalp EEG recordings at rest^72^ and during cognitive activity^73,74^. Specifically, we filtered the reconstructed regional time series in different frequency bands (delta: 1–4 Hz; theta: 4–8 Hz; alpha: 8–13 Hz; beta: 13–30 Hz and gamma: 30–45 Hz) and we applied the sliding window method in which PLV was calculated over its data points. Then PLV was averaged across sliding windows. As a result, we obtained dynamic PLVs and one static PLV in each frequency band, for each participant.

### Connectome-based predictive modeling

We employed CPM^10^ on the first dataset to construct predictive networks that can be used to estimate individual real-life creativity (C total) from resting-state EEG functional connectivity. CPM is a recently developed method for identifying and modeling functional brain connections related to a behavior of interest; real-life creativity in our case. The connectome network is then used to predict the behavior of novel participants whose data were not used in model creation. The method was previously employed and described in several studies that showed its results in predicting cognitive variables such as attention^9^ and fluid intelligence^4^. As well as to predict network alteration in several brain disorders such as sleep disorders^75^ and anxiety^76^.

**Fig. 7:**
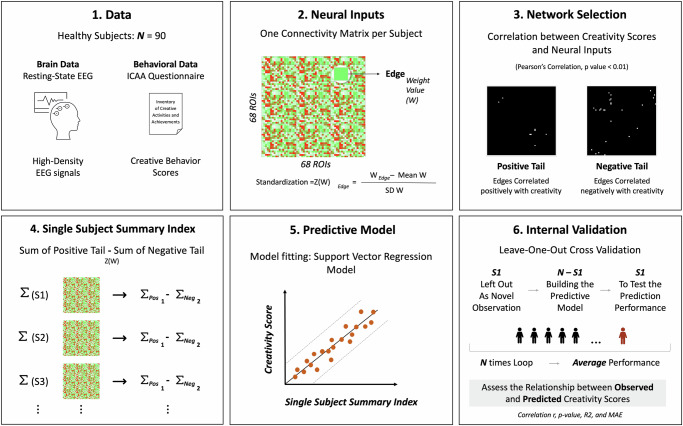
A full pipeline of the employed CPM method, as described in the “connectome-based predictive modeling” method section. **1** We employed CPM on the first dataset consisting of 90 healthy participants (*N*) to construct predictive networks that can be used to estimate individual creative behavior scores (evaluated through the Inventory of Creative Activities and Achievements or *ICAA*) from resting-state EEG functional connectivity. **2** the standardized weight values of edges in the functional connectivity matrix of each participant were calculated (for 68 Regions Of Interest or *ROIs*), where a Z-transformation was performed on each edge by calculating the difference between its weight and the mean weight divided by the standard deviation across the subjects. **3** Standardized weight values and creative behavior scores were correlated for network selection. We retained the most significant edges (*p* < 0.01) and grouped them into positive or negative tails. **4** Based on the selected networks, a single-subject summary index (*∑*) was computed for each participant (*S*) by summing the weight values of positive tail edges and subtracting the weight values of negative tail edges. **5** Then, a support vector regression (SVR) model was fitted to relate single-subject summary index and creativity scores. **6** The robustness of the model was assessed via an internal validation, where a Leave-one-out cross-validation (LOOCV) was applied. This strategy consists of removing one subject from the data as a novel observation, using N-1 subjects to build the predictive model, and then using the novel subject to test its prediction performance. This step is repeated N times with a different subject left out in each iteration (*N times Loop*). The resulting performance presents the *average* performance across all iterations. To assess the model’s predictive power, we evaluated the relationship between the observed and the predicted creativity scores using several metrics: Pearson’s correlation (*r*), parametric *p-value*, mean absolute error (*MAE*), and the coefficient of determination (*R2*).

A more comprehensive description of CPM is provided in the reference^10^. Hereafter, an overview of the processing pipeline including both training and testing procedures (Fig. 7).

First, prepare the model inputs. Two inputs were prepared: (i) the vector of behavioral values represented by creativity scores (C total) for each participant and (ii) the standardized weight values of each edge in the functional connectivity matrix of each participant. For edge standardization, as used in multiple previous studies, a Z-transformation was performed on each edge by calculating the difference between its weight and the mean weight divided by the standard deviation across the subjects in the training set^77,78^. The same parameters acquired during the training procedure (mean and standard deviation) were used to standardize the connectivity edges of the testing set.

Second, identify the predictive edges. Using Pearson’s correlation, each edge (i.e., standardized weight value) in the connectivity matrix was correlated with creativity scores, while applying a threshold (*P* < 0.01) in order to retain the most significant positively and negatively correlated edges. This step resulted in two reconstructed networks: a high-creativity network (i.e., edges correlated positively with C total scores) and a low-creativity network (i.e., edges correlated negatively with C total scores).

Third, build the predictive model. The edge strengths were computed in both positive and negative tails of correlation. By combining both creativity networks, we calculated the summed index. This latter was obtained by summing standardized weight values of all connections of the positive tail and subtracting those of the negative tail. Then, a support vector regression (SVR) model was employed to respectively relate behavioral and connectivity strength values of the training set. SVR aims to find the hyperplane that maximizes the margin between predicted and actual score values. It can handle high dimensional data advantageously and provides a non-linear model that can effectively capture complex relationships in human brain data^79,80^. Here, we used the radial basis function as a non-linear kernel.

Fourth, test the predictive model. The trained model was used to predict the creativity scores of the testing participants. Leave-one-out cross-validation (LOOCV) was applied. This strategy consists of removing one subject from the data as a novel observation and using N-1 subjects to build the predictive model, then using the novel subject to test its prediction performance. This step is repeated 90 times (i.e., the number of participants) with a different subject left out in each iteration. The resulting performance presents the average performance across all iterations.

In addition to LOOCV, we used an alternative cross-validation approach—k-fold as a dual methodological validation. This methodology involves partitioning the dataset into k equal-sized folds, with each fold serving alternately as the evaluation set while the remaining k-1 folds are used for training the model. This process is iterated k times, ensuring that each fold is used once as the evaluation set. By averaging the performance metrics obtained across the k iterations, the predictive model’s overall performance is evaluated. Based on the size of our dataset, we applied both 5 and 10 folds in our analysis.

Finally, assess the predictive model. To assess the predictive power of the established model, we evaluated the relationship between the observed behavior score and the predicted behavior score using several metrics: Pearson’s correlation (*r*), parametric *p* value (*p*), mean absolute error (MAE), and the coefficient of determination (R-squared).

Additionally, to assess the statistical significance of the prediction results for creativity networks, we performed a non-parametric permutation test by randomly shuffling the creativity scores of all participants 5,000 times. In each permutation, we randomly assigned one individual’s score to another individual’s connectivity data, repeated the LOOCV or k-fold procedure, and calculated the correlation measure averaged across folds. The 5000 correlation coefficients generated a null distribution of *R* values. Then, the non-parametric *p* value was calculated as the number of permutations where the prediction correlation is greater or equal to the true prediction correlation divided by the total number of permutations.

### External validation

Due to the slight differences in the resulting predictive networks in each iteration within the leave-one-out loop in the internal validation process, we defined the “final” generalized positive and negative predictive edges as those that were persistent in at least 90% of generated models. These positive and negative predictive networks derived from this internal validation (dataset 1) were then applied to a second independent dataset (dataset 2) for external validation. Incorporating an independent dataset is highly advised to establish the generalizability of the CPM model^81,82^.

First, edge z-transformation was applied using the same standardization parameters from Dataset 1. Then, FC strength was computed within high and low-creativity networks, and the trained SVR models were applied to predict the creativity score for each participant in the new sample. Here, the same assessment metrics were used to evaluate the predictive model’s performance (R, *p* value, MAE, and R-squared).

### Dynamic functional connectome

We applied the CPM method to investigate the presence of dynamic-dependent predictive features of creativity. The CPM framework expects punctual inputs, rendering the incorporation of dynamic functional connectivity matrices into CPM unviable. However, alternative approaches have been sought, by computing proxy metrics for dynamic functional connectivity and integrating them as the model input. One approach revolves around the computation of variance (σ^2^) along the concatenated dynamic functional connectivity time courses. This, in turn, quantified the temporal variability of edge strength, further enriching the CPM framework^83^. We calculated the variance of all edges (among Desikan-Killiany ROIs) for each subject, and we employed the same procedure of the CPM as for the static approach.

### Confounder analysis

To explore potential confounding variation, we correlated each co-variable with the creativity score (i.e., the dependent variable) using Pearson’s correlation for age and education, and Point-Biserial Correlation for gender. Education level was measured based on the official French education system, from 0 (without any diploma) to 5 (diploma of level BAC + 5 or more).

### Statistics and reproducibility

Statistics were performed using MATLAB (R2023b) software. The statistical analyses conducted on the data, as well as the sample sizes in each figure, were described in the respective captions. The resulting predictive models were internally and externally validated using two independent datasets of 90 and 41 healthy participants, respectively.

### Reporting summary

Further information on research design is available in the Nature Portfolio Reporting Summary linked to this article.

### Supplementary information

Supplementary Information

Description of Additional Supplementary Files

Supplementary Data 1

Supplementary Data 2

Supplementary Data 3

Supplementary Data 4

Reporting Summary

## Data Availability

The data generated during the current study (dataset 1) and relative additional details are available from the corresponding author upon reasonable request. Dataset 2 was shared by the University of Marseille in France and is available on request from co-author Véronique Paban. The source data behind predictive models’ validation illustrated in the Figures can be found in the supplementary information (Supplementary Data 1–4).

## Code availability

A MATLAB script (MATLAB version 6.5 or above) named “behavioralprediction.m”^10^ is available online at (https://www.nitrc.org/projects/bioimagesuite/). The code assumes that the input to the CPM protocol is a set of M-by-M connectivity matrices and a set of behavioral variables. The visualization tool used for making circle and glass brain plots shown in the Figures is available at (https://bioimagesuiteweb.github.io/webapp/).
