# Supplementary material for: Predicting creative behavior using resting-state electroencephalography
Source: Commun Biol. 2024 Jun 29;7:790. doi: 10.1038/s42003-024-06461-6 (PMC11217288; doi:10.1038/s42003-024-06461-6)
Supplement: Supplementary file 1 — Supplementary Information [file 42003_2024_6461_MOESM1_ESM.pdf]

Supplementary Information

	Internal Validation			
	F. Bands	*positive net*	*negative net*	*combined net*
C act	*alpha*	p > 0.1	p > 0.1	p > 0.1
	beta	0.0043 (r-)	p > 0.1	p > 0.1
	delta	p > 0.1	p > 0.1	p > 0.1
	gamma	0.08	p > 0.1	***0.003***
	theta	0.08	p > 0.1	***0.023***
C ach	*alpha*	p > 0.1	p > 0.1	p > 0.1
	beta	p > 0.1	p > 0.1	0.07
	delta	p > 0.1	p > 0.1	p > 0.1
	gamma	0.05	***0.02***	***0.0005***
	theta	p > 0.1	***0.005***	***0.018***
C total	*alpha*	p > 0.1	p > 0.1	p > 0.1
	beta	p > 0.1	p > 0.1	0.07
	delta	p > 0.1	p > 0.1	p > 0.1
	gamma	0.06	***0.01***	***0.0004***
	theta	0.09	***0.004***	***0.015***

Supplementary Table 1. Presented in this table are the p-values of the internal validation processes (LOOCV) of the predictive model in all frequency bands (F. Bands). In bold are the significant ones and highlighted in grey are the ones that remain significant after a permutation test (p-value of permutation < 0.05). net = network. N= 90 participants.

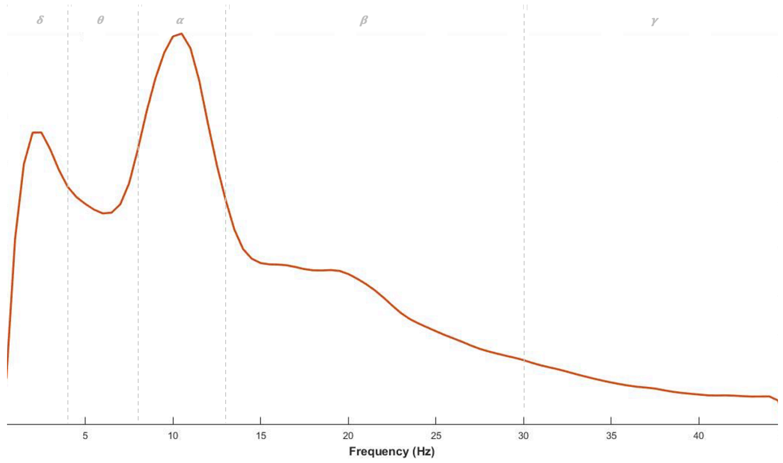

Supplementary Figure 1. This figure represents the power spectral density, averaged over all HD-EEG channels, for all subjects (0.5 to 45 Hz). With some inter-subject variability, it illustrates that alpha has the highest power followed by delta. Beta and gamma bands have lower power compared to low frequencies. Note that phase synchronization between two signals can be independent from their powers (amplitude) and brain areas with low power (such as gamma) can be strongly synchronized, and vice-versa.
