# Supplementary material for: Predicting creative behavior using resting-state electroencephalography
Source: Commun Biol. 2024 Jun 29;7:790. doi: 10.1038/s42003-024-06461-6 (PMC11217288; doi:10.1038/s42003-024-06461-6)
Supplement: Supplementary file 2 — Description of Additional Supplementary Files [file 42003_2024_6461_MOESM2_ESM.pdf]

Description of Additional Supplementary Files

File name: Supplementary Data 1

Description: The source data behind figure 2.

File name: Supplementary Data 2

Description: The source data behind figure 3.

File name: Supplementary Data 3

Description: The source data behind figure 5.

File name: Supplementary Data 4

Description: The source data behind figure 6
