# Supplementary material for: Predicting creative behavior using resting-state electroencephalography
Source: Commun Biol. 2024 Jun 29;7:790. doi: 10.1038/s42003-024-06461-6 (PMC11217288; doi:10.1038/s42003-024-06461-6)
Supplement: Supplementary file 7 — Reporting Summary [file 42003_2024_6461_MOESM7_ESM.pdf]

Corresponding author(s): Final revisions for manuscript COMMSBIO-23-2319B

Last updated by author(s): May 20, 2024

Reporting Summary

Nature Portfolio wishes to improve the reproducibility of the work that we publish. This form provides structure for consistency and transparency in reporting. For further information on Nature Portfolio policies, see our Editorial Policies and the Editorial Policy Checklist.

Statistics

For all statistical analyses, confirm that the following items are present in the figure legend, table legend, main text, or Methods section.

n/a	Confirmed	
☐	☒	The exact sample size (*n*) for each experimental group/condition, given as a discrete number and unit of measurement
☐	☒	A statement on whether measurements were taken from distinct samples or whether the same sample was measured repeatedly
☐	☒	The statistical test(s) used AND whether they are one- or two-sided<br>*Only common tests should be described solely by name; describe more complex techniques in the Methods section.*
☐	☒	A description of all covariates tested
☐	☒	A description of any assumptions or corrections, such as tests of normality and adjustment for multiple comparisons
☐	☒	A full description of the statistical parameters including central tendency (e.g. means) or other basic estimates (e.g. regression coefficient) AND variation (e.g. standard deviation) or associated estimates of uncertainty (e.g. confidence intervals)
☐	☒	For null hypothesis testing, the test statistic (e.g. *F*, *t*, *r*) with confidence intervals, effect sizes, degrees of freedom and *P* value noted<br>*Give P values as exact values whenever suitable.*
☒	☐	For Bayesian analysis, information on the choice of priors and Markov chain Monte Carlo settings
☐	☒	For hierarchical and complex designs, identification of the appropriate level for tests and full reporting of outcomes
☐	☒	Estimates of effect sizes (e.g. Cohen's *d*, Pearson's *r*), indicating how they were calculated

Our web collection on statistics for biologists contains articles on many of the points above.

Software and code

Policy information about availability of computer code

Data collection	NA
Data analysis	All MATLAB codes related to connectome-based predictive modeling is available online at https://www.nitrc.org/projects/bioimagesuite/).

For manuscripts utilizing custom algorithms or software that are central to the research but not yet described in published literature, software must be made available to editors and reviewers. We strongly encourage code deposition in a community repository (e.g. GitHub). See the Nature Portfolio guidelines for submitting code & software for further information.

Data

Policy information about availability of data

All manuscripts must include a data availability statement. This statement should provide the following information, where applicable:

- Accession codes, unique identifiers, or web links for publicly available datasets
- A description of any restrictions on data availability
- For clinical datasets or third party data, please ensure that the statement adheres to our policy

The dataset (dataset 1) generated during the current study and/or relative additional details are available from the corresponding author upon reasonable request. Dataset 2 was shared by the University of Marseille in France and is available on request from co-author Véronique Paban.

Research involving human participants, their data, or biological material

Policy information about studies with human participants or human data. See also policy information about sex, gender (identity/presentation), and sexual orientation and race, ethnicity and racism.

Reporting on sex and gender	A total of 98 healthy participants were recruited (60 females). Sex was determined based on on self-reporting (biological attribute). To explore potential confounding variation, we correlated sex with the the dependent variable (creativity scores) using Point-Biserial Correlation. Results showed no significant relation (p= 0.7). Consequently, no confounder effect was detected and thus considered as a variable in the predictive model.<br>Each participant provided written informed consent for sharing anonymously of this data.
Reporting on race, ethnicity, or other socially relevant groupings	All participants were French native speakers to avoid language confounding variation in the used verbal tasks. It has been proven that language affect brain networks. There is non-verbal non-dependent of language tests of creativity, but in this study, we used a verbal task that was proven to be the more effective evaluation of creativity. So native language was more the socially relevant variable more than race. Native language was self reported by each subject and only French native speakers were selected ("someone who has spoken a particular language since they were a baby, rather than having learned it as a child or adult").
Population characteristics	All participants were French native speakers, aged between 18 and 68 years, and with normal or corrected vision. They reported no situation or history of cognitive disability, neurological disorders, or medication that can affect the central nervous system.
Recruitment	A total of 98 healthy participants were recruited at the University Hospital Centre of Rennes from local and surrounding communities. A Flyer with Google form was lunched Online on multiple social medias, via the university site, and was send to multiple visual or performance art schools. To ensure a diverse population, participants from various creative domains such as art, dance, music, and sciences were selected. All participants were French native speakers.
Ethics oversight	The study was approved by the ethical committee of the University Hospital Centre of Rennes (agreement nº20-171)

Note that full information on the approval of the study protocol must also be provided in the manuscript.

Field-specific reporting

Please select the one below that is the best fit for your research. If you are not sure, read the appropriate sections before making your selection.

☒ Life sciences ☐ Behavioural & social sciences ☐ Ecological, evolutionary & environmental sciences

For a reference copy of the document with all sections, see nature.com/documents/nr-reporting-summary-flat.pdf

Life sciences study design

All studies must disclose on these points even when the disclosure is negative.

Sample size	Our study was exploitative, statistical tests to determine the sample size were not applicable. We determined the size from data obtained via previous experiments in our laboratory, and from the scientific literature. Our sample size was relatively larger than usual sample sizing in the literature.
Data exclusions	Participants: Only French native speakers were selected to avoid language confounding variation in the used verbal tasks. In addition, we excluded subjects who reported a situation or history of cognitive disability, neurological disorders, or medication that can affect the central nervous system.<br>EEG Data: due to their poor signal quality, 8 participants were excluded from the first dataset and 11 from the second.
Replication	The networks derived from the internal validation analysis (dataset 1) were applied to a second independent dataset (dataset 2) for external validation to establish the generalizability of our model. The external Validation was successful.
Randomization	NA
Blinding	NA

Reporting for specific materials, systems and methods

We require information from authors about some types of materials, experimental systems and methods used in many studies. Here, indicate whether each material, system or method listed is relevant to your study. If you are not sure if a list item applies to your research, read the appropriate section before selecting a response.

Materials & experimental systems

n/a	Involved in the study
☒	☐ Antibodies
☒	☐ Eukaryotic cell lines
☒	☐ Palaeontology and archaeology
☒	☐ Animals and other organisms
☒	☐ Clinical data
☒	☐ Dual use research of concern
☒	☐ Plants

Methods

n/a	Involved in the study
☒	☐ ChIP-seq
☒	☐ Flow cytometry
☒	☐ MRI-based neuroimaging

Plants

Seed stocks

Report on the source of all seed stocks or other plant material used. If applicable, state the seed stock centre and catalogue number. If plant specimens were collected from the field, describe the collection location, date and sampling procedures.

Novel plant genotypes

Describe the methods by which all novel plant genotypes were produced. This includes those generated by transgenic approaches, gene editing, chemical/radiation-based mutagenesis and hybridization. For transgenic lines, describe the transformation method, the number of independent lines analyzed and the generation upon which experiments were performed. For gene-edited lines, describe the editor used, the endogenous sequence targeted for editing, the targeting guide RNA sequence (if applicable) and how the editor was applied.

Authentication

Describe any authentication procedures for each seed stock used or novel genotype generated. Describe any experiments used to assess the effect of a mutation and, where applicable, how potential secondary effects (e.g. second site T-DNA insertions, mosiacism, off-target gene editing) were examined.
